# Developing the Disaster Medical Responder’s Course in Singapore

**DOI:** 10.5365/wpsar.2023.14.6.1009

**Published:** 2023-11-30

**Authors:** Jen Heng Pek, Li Juan Joy Quah, Kuan Peng David Teng, Yi Wen Mathew Yeo, Chan Yu Jimmy Lee

**Affiliations:** a Disaster Site Medical Command, Ministry of Health, Singapore.; b Department of Emergency Medicine, Sengkang General Hospital, Singapore.; c Department of Emergency Medicine, Singapore General Hospital, Singapore.; d Department of Emergency Medicine, Tan Tock Seng Hospital, Singapore.; e Department of Emergency Medicine, Khoo Teck Puat Hospital, Singapore.; f Department of Emergency Medicine, Ng Teng Fong Hospital, Singapore.

## Abstract

**Problem:**

Emergency medical teams (EMTs) deployed to mass casualty incidents (MCIs) are required to work outside their usual settings and according to different principles, which may affect their performance and the survival of casualties. Prior to 2013, training offered to domestic EMTs was limited to ad hoc and infrequent simulation exercises.

**Context:**

Domestic EMTs are activated from public tertiary hospitals to provide pre-hospital medical support to the Singapore Civil Defence Force and establish a first-aid post (FAP) for triaging, stabilizing and treating casualties. These casualties are then evacuated to public hospitals for further management.

**Action:**

Recognizing the need for a more systematic approach to the training of domestic EMTs, the Disaster Medical Responder’s Course (the Course) was developed as a multiinstitutional collaboration to equip EMT members attending a MCI with the necessary skills to perform effectively at the FAP.

**Outcome:**

The Course was first run in 2013 and is usually offered six to eight times a year. Since June 2019, a total of 414 health-care staff and allied health professionals have participated. There have been numerous revisions of the course content and delivery to reflect the latest concepts in operations and global best practice, as well as developments in educational methodologies.

**Discussion:**

Preparedness is crucial to optimize the survival and outcomes of casualties. The Course provides standardized training of domestic EMTs and plays a pivotal role in ensuring operational readiness for MCIs in Singapore.

## PROBLEM

A mass casualty incident (MCI) is an incident in which resources and capabilities of a health-care system run the risk of becoming overwhelmed by the number and severity of casualties. (*1*) Casualties are triaged and, as appropriate, stabilized and treated by emergency medical teams (EMTs) at the MCI site before being evacuated to a hospital, as per the “scoop and run” principle. MCIs present a unique set of challenges, often different to those faced by medical staff during the course of their routine clinical practice in a hospital environment. EMT members need to be able to adapt their roles and work processes to meet the demands of providing emergency care at the site of a MCI to improve their performance and the survival of casualties.

Prior to 2013, preparation for response to MCIs in Singapore was primarily focused on developing standard operating procedures. Training of domestic EMTs was mostly conducted during ad hoc and infrequent simulation exercises for anticipated MCIs. As Singapore is generally free from natural disasters, the training focused on responses to intentional and unintentional man-made events such as transportation accidents and building collapses, as well as explosions and releases of dangerous chemicals. The exercises often lacked clear training objectives and specific learning outcomes. Concerns had also been raised by the Ministry of Health, the Singapore Civil Defence Force (SCDF) and EMT members themselves about Singapore’s MCI operational preparedness and readiness.

In response to these concerns, the Disaster Medical Responder’s Course (the Course) was developed in 2013 to provide regular, standardized training for domestic EMT members on disaster medical response and on the role and expectations of a domestic EMT in the event of a MCI in Singapore. The primary aim of the Course was to improve the effectiveness and efficiency of the EMT response upon activation. In this report, we describe the development of the Course and how it has evolved over time.

## CONTEXT

The SCDF is responsible for delivering pre-hospital emergency medical services during peacetime and MCIs. During peacetime, SCDF is activated by calling 995, and an ambulance crew, comprising a paramedic and emergency medical technician, is dispatched to respond to medical emergencies. In the event of a MCI, the Ministry of Health, through the Disaster Site Medical Command (DSMC), supports the SCDF in their pre-hospital response by sending EMTs to the site.

All eight public tertiary hospitals in Singapore have a nominated domestic EMT comprising two doctors and four nurses rostered on every shift. Upon MCI activation, each hospital sends its EMT to the MCI site. The EMT brings its logistics bags, which contain medical equipment, drugs and consumables. They report to the DSMC and provide pre-hospital medical support to the SCDF according to the scoop and run principle. The EMTs only respond to MCIs within Singapore – they are not deployed overseas as international EMTs.

## ACTION

### Course content

The goal of the Course is to enhance disaster preparedness and operational readiness of domestic EMTs by familiarizing members with the functions and operations of a first-aid post (FAP). The learning objectives are based on Bloom’s taxonomy (levels of remember, understand and apply; **Table 1**). (*2*) The Course is run over 1 day but uses the “flipped classroom” approach, whereby the lecture slides are sent out 2 weeks beforehand so that participants can go through them before attending the Course. The Course itself consists of a combination of interactive lectures (first half of the day) and three skills stations (second half of the day). The latter comprise a communication exercise on the use of radio sets, a familiarization exercise (with the equipment and supplies available in logistics bags), and a session on the treatment of casualties using the available equipment and supplies.

**Table 1:** Learning objectives of the Disaster Medical Responder’s Course in Singapore

Learning objectives	
•	Describe the mass casualty response in Singapore
•	Discuss the command structure, roles and responsibilities of Disaster Site Medical Command and EMTs
•	Explain the activation process of Disaster Site Medical Command and EMTs during a civil emergency
•	Describe the organization of, and operations at, the first-aid post
•	Demonstrate how casualties are triaged and evacuated at the first-aid post
•	Recognize and locate the medical logistics available
•	Demonstrate the effective use of communication equipment at the first-aid post
•	Describe the psychological support available
•	Execute a table-top exercise involving the Disaster Site Medical Command and EMTs at the disaster site

EMT: emergency medical team.

The development of the course content was guided by a global operational learning framework for disaster education and training of EMTs, which has three components: (*3*)

ensuring professional competency with licence to practise;
adapting professional capacities (technical and non-technical) to low-resource and emergency contexts; and
preparing effective performance as a team in the field.

The principles and standards enshrined in the World Health Organization’s (WHO’s) *Classification and minimum standards for emergency medical teams* (2021) (the “Blue Book”) (*4*) were also considered in the development of the Course. However, as Singapore’s EMTs are intended for local deployment, only those principles and standards relevant to local concepts of operations were incorporated. For example, the Blue Book’s clinical care technical standards on triage, assessment, resuscitation, stabilization, referral and transfer, which help to improve the quality of care provided to casualties at the FAP according to the scoop and run principle, are relevant to the Course and were thus incorporated. Course content on the treatment of wounds, burns, fractures and limb injuries, as well as the use of analgesia and anaesthesia, was also based on the standards specified in the Blue Book. Similarly, WHO’s technical standards relating to medical stock management and pharmacy supply underpinned the skills station on the familiarization with equipment and supplies available in the logistics bags.

To ensure it remains relevant to current operations and aligns with best practices globally, the content of the Course has been reviewed annually by the training branch of DSMC. This process includes a thorough review of the published literature and conference proceedings.

### Course faculty

The course faculty comprises DSMC representatives nominated by their public tertiary hospitals. All faculty members are certified emergency physicians and nurses with a strong interest and training in disaster medicine and all have contributed to course content development and delivery.

### Course evaluation

Course evaluation is based on the Kirkpatrick Model (*5*) and comprises several mechanisms. Feedback is provided by faculty members during the skills stations, in the form of observations about participants’ performance in relation to the learning objectives of the Course. (*6*) For example, during the communication exercise on the use of radio sets, participants are required to demonstrate effective use of communication equipment at the FAP. Faculty members make observations about aspects of participants’ communication skills, such as clarity, simplicity and brevity of messages and use of alphanumeric phonetics, as well as the discipline of radiocommunications, which facilitates discussion with participants about their performance, the challenges they faced and potential strategies that could be used to overcome them.

A feedback form is used to evaluate the reactions of participants to the course content, the trainers and administration. Learning of the participants is evaluated through a table-top exercise based on a bomb blast scenario, conducted at the end of the Course. As part of this table-top exercise, participants assume their respective roles within the various FAP areas and are encouraged to work collaboratively to apply the concepts learned during the Course.

To evaluate behaviour change, which is indicative of sustained knowledge, skills and attitudes beyond the Course, and to maintain the competency of domestic EMTs, EMT members are invited to participate in formal exercises within Singapore. (*5*) These formal exercises are held three or four times per year and offer EMT members the opportunity to hone and refresh their skills. These formal exercises are evaluated using an exercise assessment matrix that mirrors the objectives of the Course and that provides an evaluation of the Course and an indication of the operational readiness of the EMTs. This allows gaps in training and performance to be identified and appropriate modifications to be made to improve the content and delivery of the Course.

### Education methodologies

Course development was guided by Kern’s model for curriculum development. (*7*) A literature review was performed to identify training needs and perceptions and readiness of staff involved in mass casualty responses, as well as to define expected roles, competencies and skills. (*8*, *9*)

The Course is based on the adult learning principles of i) experience of participants, ii) use of problem-centred approaches to course delivery, and iii) relevance to participants. (*10*) There is emphasis on tailoring the MCI-specific content to the current experiences of participants during non-emergencies, and allowing participants to practice what they learn during the skills stations. The Course is relevant to participants as they can apply what they have learned to direct casualty management at a FAP during a MCI.

The Course is also anchored in Gagne’s Theory of Instructional Design, which states that participant outcomes must be determined before constructing and tailoring the instructional events. (*11*) The learning outcomes (**Table 1**) were first developed by the faculty so that the curriculum would enhance the preparedness and readiness of domestic EMT members. Kolb’s learning style and experiential learning cycle were also applied to enhance learning through participant reflection on their practice and sharing of experiences during the interactive lectures and skills stations. (*12*) By adopting these approaches, participants are encouraged to observe, think, experiment and perform as part of their learning process.

The use of simulation exercises, which are integral components of the skills stations and the final table-top exercise, provides experiential learning to participants in a safe setting. (*13*) Simulated aides are used to replicate real-life MCI casualties, and participants are required to make clinical decisions about the management of these casualties at the FAP.

## OUTCOME

Since 2013, the Course has been run between six and eight times per year. From June 2019 onwards, the Course was conducted at a single site, the SingHealth Duke-NUS Institute of Medical Simulation; previously it was held at various public hospitals on a rotational basis. A total of 414 participants have attended 14 courses since June 2019 (**Table 2**).

**Table 2:** Disaster Medical Responder’s Course participants by role and workplace, Singapore, June 2019 to December 2022 (*n* = 414)

Role	No. of participants, *n*(%)
Doctors	113 (27.3%)
Nurses	256 (61.8%)
Allied health professionals	8 (1.9%)
Administrative staff	37 (8.9%)
**Workplace**	
Public hospitals	354 (85.5%)
Other health-care institutions	57 (13.8%)
Ministry of Health	3 (0.7%)

The Course was last updated in 2020 in response to the coronavirus disease pandemic. Introduction of safe management measures in Singapore, such as minimizing in-person gatherings and limiting group sizes, led to modifications to course delivery. Interactive lectures were delivered via an online platform, participants were divided into smaller institutional groups for the skills stations, and the individual groups were assigned the same faculty member. Additional cleaning requirements increased the duration of the Course and the amount of administrative support needed.

## Discussion

In this report, we have described the development of the Disaster Medical Responder’s Course, which aims to enhance disaster preparedness and operational readiness of domestic EMTs responding to MCIs in Singapore. Our experience and lessons learned in developing this training course may be useful for other countries wishing to create a similar course specific to their context.

The short 1-day duration of our face-to-face course was made possible by the use of a flipped classroom approach, whereby the theoretical content is delivered ahead of the in-person part of the Course. (*14*) This approach allows participants to view the required materials for knowledge acquisition at their own pace and in an environment of their choice before attending the in-person part of the Course, which can then be devoted to interactive discussions and skills stations. As well as reducing in-person course time, the flipped classroom approach has been shown to improve student satisfaction and engagement with learning. (*14*)

Due to its practical nature, the use of a table-top exercise to evaluate the Course was preferred over more theoretical assessments (e.g. multiple-choice tests) and also over a large-scale simulation exercise. A table-top exercise exposes participants to operations at the disaster site in a step-wise manner, and allows them to apply what they have learned at each stage. A large-scale simulation exercise is staff- and resource-intensive, making it a less sustainable and cost-effective option for the evaluation of a 1-day course. However, formal full-scale exercises that build on the 1-day course evaluation process are conducted routinely each year.

The course content and delivery are similar to other training courses that have been developed across the Western Pacific Region for other EMTs such as Japan’s Disaster Medical Assistance Team (DMAT), the Republic of Korea’s DMAT, the Australian Medical Assistance Teams (AUSMAT) and the New Zealand Medical Assistance Team (NZMAT). These similarities exist despite the fact that these national EMTs are designed for international deployment, whereas Singapore’s EMT has a domestic deployment focus. In terms of the content, commonalities exist in the concept of operations, command and control, in the roles and responsibilities of team members, and in essential field skills such as triage, medical management, logistic familiarization and communications. Methods of delivery generally include a mix of didactic or interactive lectures, practical trainings and simulations including desktop and table-top exercises.

The move to a centralized delivery model in 2019 proved advantageous on several levels. It meant that the faculty and participants were drawn from different public tertiary hospitals, whereas previously the faculty and participants were restricted to individuals from the public tertiary hospital hosting the Course. A centralized delivery model not only allows more diverse interaction and exchange of experience and practice but also replicates the real-world MCI setting in which EMT members from different public tertiary hospitals have to work together. In addition, a dedicated, single course location simplifies the logistics of delivering a training course and is therefore more cost-effective. The centralized model also widens participation and increases the number of participants that can be trained. However, the varied background of participants can pose a challenge, requiring the faculty to tailor the content and delivery of the Course to maximize attendees’ learning.

There are some limitations to this report. As there have not been any MCIs within Singapore that required deployment of domestic EMTs, we were unable to assess how the training has contributed to real-world preparedness and response. Also, we were unable to analyse course evaluations that were conducted before the centralization of the Course in 2019, and therefore information relating to course evaluation is limited to that from June 2019 onwards.

In conclusion, ensuring preparedness of domestic EMTs is a vital part of disaster response. It requires a continuous cycle of planning, organizing, training, equipping, conducting exercises and evaluations, and making adjustments to ensure that EMTs responding to a MCI can function to their maximum capacity and capability and thereby optimize the survival of casualties and their outcomes. (*15*)
